# Utilization of long-acting contraceptive methods and associated factors among married women in Farta Woreda, Northwest Ethiopia: a community-based mixed method study

**DOI:** 10.1186/s12905-022-02092-3

**Published:** 2022-12-19

**Authors:** Eden Workneh Aychew, Yibeltal Alemu Bekele, Alemu Degu Ayele, Anteneh Mengist Dessie, Gizachew Worku Dagnew

**Affiliations:** 1 Department of Midwifery, College of Health Sciences, Debre Tabor University, Debre Tabor, Ethiopia; 2 Department of Reproductive Health and Population Studies, School of Public Health, Bahir Dar University, Bahir Dar, Ethiopia; 3 Department of Public Health, College of Health Sciences, Debre Tabor University, Debre Tabor, Ethiopia

**Keywords:** Long-acting, Contraceptive, Married women, Farta district

## Abstract

**Background:**

Although long-acting contraceptive methods are highly effective, safe, and provide uninterrupted protection from unintended pregnancy compared to short-acting methods, evidences suggest that majority of women were relayed on short-acting contraceptive methods. Thus, this study aimed to determine the level of long-acting contraceptive methods utilization and associated factors among married women in Farta Woreda, Northwest Ethiopia, 2021.

**Methods:**

A community-based cross-sectional study supplemented with qualitative data was performed among 556 married women from March 1 to 31, 2021. A semi-structured interviewer-administered questionnaire and interview guides were used to collect the data. The data were entered into Epi data version 4.6 and analyzed by SPSS version 23 software. The association between variables was analyzed using bivariate and multivariable binary logistic regression. The level of statistically significant association was determined at a *P*-value < 0.05. After translation and transcription, manual thematic analysis was applied to the qualitative data.

**Results:**

The magnitude of long-acting contraceptive methods among married women in Farta Woreda was found to be 14.3% [95% CI 11.5–17.6]. Previous use of long-acting contraceptive methods (AOR = 5.83, 95% CI 3.03, 11.21), positive attitude towards long-acting contraceptives (AOR = 2.74, 95% CI 1.48, 5.07), having formal education for husbands’ (AOR = 3.05, 95% CI 1.5, 6.21), and poor wealth index (AOR = 3.39, 95% CI 1.33, 8.67) were positively associated with utilization of long-acting contraceptive methods. Moreover, fear of side effects, misconceptions, and partner opposition were the most commonly identified barriers by the qualitative data.

**Conclusion:**

Utilization of long-acting contraceptives among married women in Farta Woreda was low as compared to the 2020/21 national reproductive health strategy plan to increase the long-acting reversible and permanent contraceptive methods use to 50%. Previous use of long-acting contraceptives, positive attitude towards long-acting contraceptives, husband education, and household wealth index was found to be significantly associated with long-acting contraceptive utilization. Hence, it is better to work more on changing women's attitudes and increasing husband education.

## Plain English summary

Long-acting contraceptive methods (LACM) are methods used to prevent pregnancy for a longer period than short-acting methods. LACM are classified in two categories, reversible and non-reversible (permanent).

This study use a community-based quantitative cross-sectional study supplemented with qualitative data among married women from March 1 to 31, 2021 in Farta Woreda northwest Ethiopia. The association between variables was analyzed using bivariate and multivariable binary logistic regression. Statistically significant association was declared at a *P*-value < 0.05. For the qualitative data, a thematic analysis was made manually.

The current study found that the magnitude of long-acting contraceptive methods among married women in Farta Woreda was 14.3% 95% CI [11.5–17.6]**.** Women’s husband education, wealth index, previous use LACM, and attitude were significantly associated with long-acting contraceptive utilization. Besides, fear of side effects and partner opposition were the most common barriers of LACM.

This study report that utilization of long-acting contraceptives among married women in Farta Woreda was significantly low as compared to the national reproductive health strategy 2020/21 plan. Therefore, establishing strategies and polices to change women’s attitudes and increasing husband education will be essential when designing implementation plans to scale up uptake of LACM.

## Background

Long-acting contraceptive methods (LACM) are highly effective, safe, cost effective, and provide uninterrupted protection for 3 to 12 years to prevent unwanted and unintended pregnancy than short-acting contraceptive methods [1].

Long-acting contraception methods can be classified in two categories, either reversible or non-reversible (permanent). Hormonal implants (preventing pregnancy for 3–5 years) and Intra-Uterine devices (either non-hormonal or hormonal; preventing pregnancy for 5–10 years) are under reversible contraceptive methods. Whereas tubal-ligation (female sterilization) and vasectomy (male sterilization) are non-reversible or permanent methods [2].

In addition to its effectiveness, safety, and cost effective, LACM had extra non-contraceptive health tips including reduction of menstrual bleeding in patient with menorrhagia (levonorgestrel-releasing intrauterine system (LNG-IUS)), premenstrual mood disturbance, and endometrial cancer (Copper IUD) [3–5].

In developing regions, nearly 214 million reproductive age group women have unmet need for modern contraceptive methods. Due to this almost half of the pregnancies were unintended [6]. Besides, one in every three unintended pregnancies resulted from contraceptive failure, which was highly prevalent in short-acting and traditional methods compared with long-acting contraceptive methods. (4.6–22% vs 0–1.8%) respectively [7].

Ethiopia is the second most populous African country next to Nigeria affected with fast population growth [8]. However, according to 2016 EDHS report 22% of married women still has an unmet need for family planning [9].

Providing affordable, acceptable, and available contraceptive methods is important to prevent unintended pregnancy and to help families and countries to achieve their health goals [7]. Meanwhile, evidence suggests that providing effective family planning (FP) interventions contributed to more than a 25% reduction in the maternal mortality ratio [10].

Globally around 45.2% of women residing in developed world are utilizes long-acting contraceptive methods [11]. However, the utilization of these highly effective and safe LACM is significantly low in developing countries including Ethiopia [12–15].

In the past decades, different initiatives such as Marie Stops International (MSI) and Population Services International (PSI) are advocating the utilization of LACM to increase its access and demand [16]. The 2020 FP plan has an agenda to address 120 million additional modern contraceptive users in the world’s least-developed countries improved access and demand to long-acting contraceptive methods [17].

The Government of Ethiopia is highly dedicated to achieve Sustainable Development Goal three (SDGs-3), and strongly believed that FP is one of the key strategies for improving maternal health by minimizing unwanted and unintended pregnancies and their complications. In this regard the revised National Reproductive Health Strategy (2016–2020/21) of the Federal Ministry of Health (FMOH) of Ethiopia worked hard to scale up the utilization of LACM by 50% in the year 2021 [18].

In Ethiopia different study findings including the Mini Ethiopian Demographic Health Survey (MEDHS) 2019 show that the utilization of long-acting contraceptive methods is still very low ranging from 11 to 26.4% indicated that the target of increasing LACM to 50% is not met; as a result extra effort and studies are needed to tackle the problem [19–24].

However, there is no available evidence on the real magnitude and associated factors of the demand for LACM in Amhara region specifically in the study area. Hence, this study was mainly aimed to assess the magnitude of LACM utilization and its associated factors among married women in Farta Woreda northwest Ethiopia. Besides this study tried to identify barriers that hinder the utilization of LACM in the community using a qualitative approach.

## Methods

### Study design and setting

A Community-based cross-sectional study supplemented with qualitative data was conducted from March 1–30, 2021 in Farta district. Farta district/woreda is found in the south Gondar zone of Amhara regional state Northwest Ethiopia. It is located around 585 km away from Addis Ababa (the capital city of Ethiopia), and 102 km from Bahir Dar (the capital city of Amhara national regional state). The woreda is divided in to 33 small administrative units called kebeles with an estimated total population of 230,731 based on the 2020 report obtained from the woreda administrative [25]. Among the total population, the reproductive-age women account for 54,406. In the woreda, there are 3 private clinics, 8 health centers, and 36 health posts providing FP services for the community.

### Population

The source population was all reproductive age group married women who were living in Farta district and the study population comprises reproductive age group married women who were living in selected kebeles of the district during the study period. Reproductive age group married women who had hysterectomy and resided less than six months were excluded from the study. For qualitative study, women with history of LACM use, health extension workers who provide more LACM and women developmental army leaders with advanced age were included.

### Sample size determination and sampling procedure

A sample size of 556 participants was determined by using the single proportion population formula with the assumption of the utilization of (proportion) LACM from previous study done in Adama was 20.9% [23], 95% confidence interval, 5% margin of error, design effect of 2 (as two stage of sampling techniques was carried out to select the participants), and adding a 10% non-response rate.

A multistage sampling technique was used to select the study participants. Initially we select representative kebeles using simple random sampling technique. In the second stage, we used computer generated simple random method to select the households. Out of the total 33 kebeles, nine kebeles were selected using the lottery method. Households with married women of selected kebeles were obtained from respective health post family folder. Accordingly there were a total of 12,310 women who were utilizing LACM in selected kebeles. Then a sampling frame was generated containing these 12,310 married women. Finally, the calculated 556 study participants were selected through computer generated simple random sampling technique starting from kebele one from a random start point.

One married woman per household was selected. If two or more eligible women were encountered in one household, only one was included using lottery method and if no eligible women were identified in the selected household, the next nearest eligible household located in a clockwise direction was visited until we achieved the desired sample size. For qualitative data, the heterogeneous (maximum variation) type of purposive sampling technique was employed to select the participants until data saturation was reached. LACM users and non-users, husbands of LACM user and non-user, health extension workers, and HDA leaders were included in the qualitative study and quantitative study participants were not part of the qualitative section.

### Variables

#### Dependent variable

Utilization of Long-acting contraceptive method.

#### Independent variables

*Socio-demographic characteristics* (Age, Education status, Occupation, Religion, Wealth index, Partner educational status).

*Reproductive health-related characteristics* (Future desired number of children, number of alive children, gravidity, parity, abortion history, family size, previous use of LACM, history of unwanted pregnancy, number of under-five children).

*Individual characteristics* (Knowledge of LACM, Attitude towards long-acting contraceptive methods, Discussion with husband about LACM).

*Physical accessibility of family planning service* (Distance).

### Operational definitions

#### Long-acting contraceptive methods

Are contraceptive methods which comprises both reversible (implants and intra-uterine device) and non-reversible (tubal ligation) methods.

#### LACM utilization

Women who are currently (at the time of data collection) using long-acting contraceptive methods [26].

#### Knowledge

Knowledge of married women about LACM was measured using five knowledge assessing questions. A value of one and zero was given for each correct and incorrect response respectively. Those women who scored above the mean score were considered as knowledgeable and those women who scored mean score and below the mean score were considered as not knowledgeable for LACM [27].

#### Attitude

The attitude of women towards LACM was assessed by using ten negatively stated attitude related questions, and maximum score was given when participants strongly disagree for the questions and lower points when they strongly agree and labeled as positive attitude for those participants who scored greater than the mean score and negative attitude for those participants who scored mean score and below the mean score [28].

#### Physical access to health service

Services are considered to be physically accessible if the distance from women's home to the health facility where they get access to FP is takes less than 30 min by foot [29]. It is assessed by asking the women the average time it takes from her home to the facility where she uses family planning methods.

### Data collection tool and quality management

The quantitative data were collected via interviewer administered approach by using semi-structured and pretested questionnaires. The tool was first prepared in English after reviewing different literature, then translated to the local language Amharic, for simplicity, and back to English to maintain its consistency by different language experts who speak both English and Amharic fluently. Pre-testing of the tool was conducted on 5% of participants (28 married women) in one non-selected kebele. Based on the pre- test finding, immediate modifications and corrections like wording, logical sequence and skip patterns were corrected before used for the actual data collection. The data were collected by nine diploma health worker and supervised by three BSc health professionals. Data collectors and supervisors were trained for one day duration on the aim of the study, method of data collection, and content of questionnaire. The qualitative data were collected using in-depth interviews by two individuals who have previous experience until information saturation was reached. The data were saturated with six in-depth interviews and four key informants’ interviews.

### Data processing, analysis, and interpretation

The collected data were entered into Epi-Data version 4.6 and then exported to SPSS version 23 for analysis. Descriptive statistics like frequencies and percentages were used to present the categorical independent variables and mean and standard deviation was used to describe a continuous variable. Tables and graphs were used to present descriptive results.

Bivariate logistic regression analysis was executed by computing odds ratio (OR) with a 95% confidence interval to see the association between each independent and dependent variable. Finally, all independent variables associated with dependent variables with *p* < 0.2 were fitted into multivariable logistic regression for further analysis, and lastly the significant association was declared on *p* < 0.05 with 95% CI.

For the qualitative data, a thematic analysis was carried out. First, the recorded data was listened to repeatedly and it was translated word by word into text using the local language. Then transcribed verbatim from the local language into English and presented in narratives by using participants own words. After developing codes, all the issues discussed under each idea were collected in to three main themes. Finally, the data were analyzed manually according to the identified thematic areas.

*Declarations* All methods were performed in accordance with the relevant guidelines and regulations and Bahir Dar University college of medicine and health science institutional review board had given approval for the study with the protocol number 078/2021.

## Results

### Socio-demographic characteristics

A total of 544 married women participated in the study making a response rate of 97.8%. The mean age of the participants was 32.97 (SD ± 5.89) years with a predominant age group of 25–34 years (49.4%). Almost all of the study participants were Amhara by Ethnicity and Orthodox Christian religion followers. Among the participants, 435 (80.0%) had never attended any formal education. All households included in the study were male-headed and 514 (91.7%) participants were housewives. Regarding the household wealth status, 204 of the participants were found in the lowest wealth tertile (poorest) (36.1%) and two 213 of them were in the middle class (37.7%) (Table 1).

**Table 1:** Socio-demographic characteristics of study participants in Farta District Northwest Ethiopia, March 1–30, 2021 (n = 544)

Variables	Frequency	Percent (%)
*Age category*		
15-24 years	46	8.5
25–34 years	269	49.4
> 35 years	229	42.1
*Educational status*		
No formal education	435	80
Have formal education	109	20
*Husband educational status*		
No formal education	418	76.8
Have formal education	126	23.2
*Occupation*		
House wives	498	91.5
Daily laborer	37	6.8
Merchant	4	0.7
Government employee	5	0.9
*Husband occupation*		
Farmer	443	81.4
Merchant	39	7.2
Daily laborer	36	6.6
Government employee	26	4.8
*Family size*		
1–5	245	45
6–10	299	55
*Wealth index*		
Poorest	222	40.8
Middle class	168	30.9
Richest	154	28.3

### Reproductive health characteristics

Of the total study participants, 333 (61.2%) and 167 (30.7%) were multigravida (two and more pregnancies) and grand multigravida (five and more pregnancies) respectively. In this study, the study participants had an average of four live children with a standard deviation of (SD ± 1.85). 374of the participants (68.8%) want to have more children in the future and the average future desired number of children was two with (SD ± 0.79). Nearly all study respondents (99.8%) had a history of previous pregnancy and more than three-fourth 447 (79.1%) of the participants had a history of ANC follow-up for their last pregnancy (Table 2).

**Table 2:** Reproductive health characteristics of participants in Farta Woreda Northwest Ethiopia, March 1–30, 2021 (n = 544)

Variable	Frequency	Percent (%)
*Gravidity*		
Nulligravida	1	0.2
Primigravida	43	7.9
Multigravida	333	61.2
Grand multigravida	167	30.7
*Parity*		
Nulliparous	11	2.0
Primiparous	42	7.7
Multiparous	366	67.3
Grand multiparous	125	23.0
*History of abortion*		
No	387	71.1
Yes	157	28.9
*History of stillbirth*		
No	482	88.6
Yes	62	11.4
*Live children*		
0–4	340	62.5
> = 5	204	37.5
*A future desire for more children*		
No	170	31.3
Yes	374	68.8
*Decision on number of children*		
My self	10	1.8
My husband	121	22.2
Together and with others	413	76
*Previous use of LACM*		
No	309	56.8
Yes	235	43.2

### Individual characteristics

In this study, 284 (52.2%) and 269 (49.4%) of the study participants were knowledgeable and found to have a positive attitude towards long-acting contraceptive methods respectively. Implants were the most popular long-acting contraceptive methods known by the participants whereas vasectomy was the least known contraceptive methods (3.3%). Health extension workers were the major source of information regarding LACM for the study participants (97.1%) (Table 3).

**Table 3:** Individual characteristics of participants in Farta Woreda Northwest Ethiopia, March 1–30, 2021 (n = 544)

Variables	Frequency	Percent
*Knowledge*		
Not knowledgeable	260	47.8
Knowledgeable	284	52.2
*Attitude*		
Unfavorable attitude	275	50.6
Favorable attitude	269	49.4
*Discussion about FP with partner*		
No	110	20.2
Yes	434	79.8
*Source of information*		
Mass media	194	35.7
Family and friend	141	25.9
Health institution	406	74.6
Health extension worker	528	97.1

### Utilization of long-acting contraceptive methods

The overall utilization of LACM was 14.3%, with a 95% CI of [11.5, 17.6]. Majority of the participants, 75 (96.2%) were utilized implants while the rest were utilized tubaligation (female sterilization). This study identified that fear of side effects and complication were the most dominant reason for not utilized LACM accounts 282 (60%) followed by partner opposition mentioned by 130 (27.9%) study participants.

### Factors associated with utilization of LACM

Bivariate logistic regression analysis was performed for all independent variables to identify candidate variables for the multivariable regression. Women education, husband’s education, family size, number of live children, wealth index, future desire of more children, history of abortion, previous use of LACM, discuss family planning options with husband, knowledge, and attitude were found to be the candidate variable for multivariable analysis at *P*-value < 0.2. On multivariable logistic regression analysis, husband education, household wealth index, previous use of LACM, and attitude towards LACM were significantly associated with utilization of LACM.

Women whose husbands had attended formal education were 3.0 times more likely to utilize LACM (AOR = 3.05, 95% CI 1.5, 6.21) compared with those women whose husbands did not attend formal education. Women who had the lowest tertile wealth index were 3.3 times (AOR = 3.39, 95% CI 1.33, 8.67) higher the odds of LACM use compared to their counterparts. Similarly, participants who had second tertile household wealth index were 3.0 times (AOR = 3.06 95% CI 1.28, 7.32) more likely to utilize LACM than participants who had upper household tertile. Moreover, study participants who had a previous history of LACM use were nearly 6.0 times (AOR = 5.83, 95% CI 3.03, 11.21) higher the odds of service utilization compared to those participants who had no previous experience of LACM utilization. Lastly, women who had positive attitude towards LACM were nearly 3.0 times (AOR = 2.74, 95% CI 1.48, 5.07) more likely to utilize the service as compared to their counterparts (Table 4).

**Table 4:** Factor associated with utilization of long-acting contraceptive methods in Farta Woreda Northwest Ethiopia, March 1–30, 2021 (n = 544)

Variable	Currently utilizing LACM		COR (95% CI)	AOR (95% CI)
	No	Yes		
*Education status*				
No formal education	382	53	1	1
Have formal education	84	25	2.15 (1.26, 3.64)	0.87 (0.39, 1.93)
*Husband education*				
No formal education	378	40	1	1
Have formal education	88	38	4.08 (2.17, 6.73)	3.05(1.5,6.21) *
*Family size*				
2–5	199	46	1	1
6–10	267	32	0.52 (0.32, 0.84)	0.65 (0.28, 1.49)
*Number of live children*				
0–4 child	280	60	1	1
> = 5 child	186	18	0.45 (0.25, 0.78)	0.96 (0.4, 2.31)
*Wealth index*				
Poorest	184	38	2.97 (1.43, 6.17)	3.39 (1.33, 8.67) *
Middle class	138	30	3.13 (1.47, 6.64)	3.06 (1.28, 7.32) *
Richest	144	10	1	1
*History of abortion*				
No	338	49	1	1
Yes	128	29	1.56 (0.94, 2.58)	0.86 (0.46, 1.58)
*A desire for more children*				
No	154	16	1	1
Yes	312	62	1.91 (1.06, 3.425)	0.65(0.27, 1.57)
*Previous use of LACM*				
No	287	22	1	1
Yes	179	56	4.08 (2.41, 6.91)	**5.83 (3.03, 11.21**) *
*Discussion with partner*				
No	84	26	1	1
Yes	382	52	0.44 (0.26, 0.74)	0.71 (0.38, 1.31)
*Attitude*				
Unfavorable attitude	257	18	1	1
Favorable attitude	209	60	4.09 (2.34, n7.15)	2.74 (1.48, 5.07) *
*Knowledge*				
Not knowledgeable	236	24	1	1
Knowledgeable	230	54	2.31 (1.38, 3.86)	1.09 (0.57, 2.08)

Bold and *Statistically significant

### Qualitative findings

The description of the qualitative samples by key socio-demographic characteristics was presented here as follow (Tables 5 and 6).

**Table 5:** Key informant interview participant’s information in Farta Woreda, 2021

S.no	Participant code	Sex	Age	Occupation
1	KI 1	Female	38	HDA leader
2	KI 2	Female	42	HDA leader
3	KI 3	Female	28	Health extension worker
4	KI 4	Female	29	Health extension worker

**Table 6:** In-depth interview participant's information in Farta Woreda, 2021

S.no	Participant code	Sex	Age	Occupation	LACM utilization
1	IDIP 1	Female	25	Housewife	No
2	IDIP 2	Female	38	Merchant	No
3	IDIP 3	Female	31	Daily laborer	Yes
4	IDIP 4	Female	37	Housewife	Yes
5	IDIP 5	Male	28	Merchant	No
6	IDIP 6	Male	39	Farmer	No

To triangulate the qualitative findings, in-depth and key informant interviews findings were summarized into themes that emerged during interviews. The most repeatedly mentioned barriers by the participants were grouped into three main themes; myth and misconception, partner disapproval (opposition), and health service-related problems.

#### Myth and misconception

There are different myths and misconceptions regarding long-acting contraceptives in the community. Both in-depth and key informant interview participants mentioned that the side effects of long-acting contraceptives are not tolerable and they didn’t want to use it despite its numerous advantages. They also mentioned it is luxury to use LACM and deal with it since they had a lot to do in the house. The most repeatedly mentioned misconceptions are muscle weakness &prevention from doing different activities, needs diversified diet, infertility, and changing behavior.

A 25 years old short-acting family planning user says *“I have used Implanon before it is very bad, I couldn’t speak healthy (Selam ayawaragnem) even with my little child since it [LACM] controls my head besides. I also lost my appetite and lose much weight (kasewenate tera wetahu)”.*

Similarly, another 38 years old study participant also says “*my relative told me that she couldn’t get pregnant even if it was more than a year since she had it removed, in addition, she also says she couldn’t do her home activity because it weakens her hand (ejuwane selaselelewe)”*.

A 39 years old male in-depth interview participant also said that “they [long-acting users] *always complain their hand is weak and couldn’t manage the heavy work around the house…….in addition my wife always says she is on menses (dema aleteram), this is difficult.”*

Moreover*, 39 years old women said that “I am using depo Provera and my old sister uses Implanon which causes dizziness, irregular bleeding, body decrement, and can’t work easily with her hand due to these problems she visits the health institutions repeatedly to remove it. However, the health professionals simple counsel something and send her back to home. Finally, she went to the private clinic and removed with payment. Currently, she is health and use injectable methods like me. After these occasions I didn’t want to hear about implant.*

On the contrary, in-depth interview participants had mentioned that utilization of long-acting methods was beneficial in many ways. *Thirty-one years, old in-depth interview participant says* “*it has been a year and a half since I use Implanon, it is better than depo because I don’t have to travel to the health post every three months and worry about my appointment day*”.

#### Partner opposition

Partner opposition is the other repeatedly mentioned barrier for utilizing long-acting contraceptive methods.

*A 38 years old study participant says “my child is only 6 months old and I don’t want to be pregnant until she is 3 years old but my husband doesn’t allow me to use the long-acting method”.*

#### Health service-related barriers

The other main finding the qualitative study revealed was the health service-related barriers, which were the untruth fullness of health providers and incompetency of providers. These were the main barrier for the community not to use the long-acting method as mentioned by both key informants and in-depth participants.

*A 38 years old key informant study participant mentioned that “the main problem the community mention is that health extension workers beg and promise a lot for women to use long-acting method but they give different reasons and refuse to remove on the time she wants, so they had to go to a far by facilities or beg health providers bringing their husbands or elderlies in the community so that they [women] prefer the method they have control on it.’’.*

*A 25 years old in-depth interview participant said “when I was using Implanon, I went to the health extension worker to get removal. She [health extension worker] was convinced to remove after a lot of time. Then when trying to remove it she couldn’t find it. I bleed a lot it was painful and the wound was there for a long time”.*

## Discussion

This study found that only 14.3% with 95% CI [11.5–17.6] of participants were utilized LACM during the study period. This was consistent with a study conducted in Mekele district 12.35 [28] and Janamora district 12.9% [30]. However, it is higher than a study done in Goba town (8.7%) [31]. The discrepancy may be due to the time difference in which the study is conducted since the advocating of LACM had increased from time to time through the implementation of the package of family health and expanded training of health workers on FP [18]. On the contrary, the result of this study shows that the utilization of LACM is lower than a study conducted in Debra Markos (19.5%) [19], Nakemet (20%) [32], Adama town (20.9%) [23], Debre Birhan (21.9%) [33], and Adaba (30.3%) [34]. The possible explanation might be differences in participant’s residences. For example, study done in Nakemet, Adama and Debre Markos majority of the participant was urban residency which makes them in better accessibility of the service. Once more, urban population have easily accessed information regarding to contraceptive methods through different media [18], which minimizes the vulnerable of the participants to myths and misconception which negatively affect the uptake of contraceptives.

The current finding is also lower than two studies conducted in Iran 27.7% and 21.4% [35, 36]. This could be due to differences in the socio-demographic characteristics of the population.

The finding of this study showed that the level of contraceptive service utilization of women was linearly increases with the educational status of their husbands. One potential reason might be since males are the most influential decision-makers in our society which reaches up to 60% [37], as their educational status increased, they know the benefit of different contraceptive methods. Our study also showed that 34% of the decision regarding current contraceptive method utilization is made by husbands alone which showed that male involvement plays a significant role in utilization of contraceptive methods. This also supported by the qualitative finding which shows that the partner’s decision was also a key predictor of LACM utilization as their disapproval is one main barrier for not utilizing the service. Another potential reason might be as the women’s husband education increases, their health-seeking behavior regarding different maternal and child health services including FP will also improve as they can positively influence by their husbands [38]. Hence**,** increasing male educational level will be one strategic plan to incase male involvement in FP.

In this study, the odds of LACM utilization have an inverse relation with household wealth index. Women who have lower household wealth index are inversely proportional with level of LACM utilization. This finding was supported by a study conducted in Bangladesh, India, and Haiti [39]. But the finding is contradicted with a study finding conducted in Arba Minch district [40], which reported that wealthier participants were more likely to utilize long-acting contraceptives.

The possible explanation could be higher wealth index families may prefer many children since they can easily grow their children. Richer families also think that they can sustain their wealth with the help of their children. Once more, wealthier family’s needs more siblings to hand over their assets when they passed away. On the contrary poor families with poor wealth index are obligated to minimize their number of children’s by using LACM for effective and durable prevention of pregnancy because child birth and rearing needs more costs.

Moreover, previous history of long-acting contraceptives utilization was significantly associated with the current utilization the service. The result of this study is in agreement with a study conducted in Bahir Dar [26] and Gondar [27]. The possible explanation might be women who have previous experience of LACM can get plenty of information and counsel regarding the benefit of LACM, its advantage over short-acting methods, and costs by health care providers. Besides, women with previous exposure to LACM might give witness for the methods they used rather than considering rumors and misconceptions. The qualitative finding also supports this idea, which showed that the advantage of methods previously encourages them to utilize again in the current time.

Lastly, the current study noted that level of attitude regarding LACM had a strong relationship with utilization of LACM. Those women who had positive attitude towards LACM had an increased chance to utilize the service. The finding of this study is parallel to the study findings from Adama [23], Bahir Dar [26], Adaba [34], Arba Minch [40], Bati [41], Layarmacheho [42], East Gojjam [43], and Gondar [44]. The probable reason might be since attitude is a crucial indicator that may affects the decision-making ability of an individual towards utilization of LACM. Women with positive attitude towards LACM can break myths, rumors, and misconceptions that were negatively influencing the uptake of long-acting contraceptive methods.

Although this study was a community-based mixed methods which would help to know the real practice and dig out barriers that hinders the utilization of LACM at the community level. But it has its limitations. First, the data were entirely collected from women as a result vasectomy one of the LACM is not assessed. Second, social desirability bias might be the challenge when a woman answers the questions. Third, due to minimal sample size the generalizability of the finding might be affected and qualitative data also has its own limitation in relation to interviewing environment selection.

## Conclusion and recommendation

In conclusion, the magnitude of long-acting contraceptive methods utilization among married women in Farta Woreda was significantly low as compared to the national reproductive health strategy 2020/21 plan. Husband education, household wealth index, previous use of long-acting contraceptives, and positive attitude were found to be significantly associated with long-acting contraceptive utilization. Thus, we recommended the government, program planners, and implementers should be focused to address socio-cultural barriers, misconceptions that negatively influences attitude towards contraceptives, and increase partner involvement though education. The qualitative, data also identified that myth and misconceptions, partner opposition were the main barriers that hinders up take of LACM.

## Data Availability

The datasets used and/or analyzed during the current study are available from the corresponding author on reasonable request.

## Abbreviations

EDHS: Ethiopian demographic and health survey
FMOH: Federal ministry of health
IUD: Intra-uterine device
LACM: Long-acting contraceptive methods
SSA: Sub-Sahara Africa
